# RNA editing in Parkinson’s disease: emerging mechanisms, translational potential, and current challenges

**DOI:** 10.3389/fnins.2026.1850954

**Published:** 2026-05-15

**Authors:** Shutong Liu, An Wu

**Affiliations:** 1 Jinhua Graduate Joint Training Base, Zhejiang Chinese Medical University, Zhejiang, China; 2 Department of Neurosurgery, Quzhou People’s Hospital, Quzhou, Zhejiang, China

**Keywords:** ADAR, A-to-I editing, neuroinflammation, Parkinson’s disease, RNA editing, α-synuclein

## Abstract

Parkinson’s disease (PD) is a major neurodegenerative disorder characterized by progressive loss of dopaminergic neurons and accumulation of α-synuclein. Current treatments primarily focus on symptom alleviation, highlighting the necessity for identifying novel molecular therapeutic targets. RNA editing, as a post-transcriptional process that modifies RNA sequences without altering genomic DNA, is increasingly recognized as an important contributor to the neuronal development and synaptic regulation. Among known RNA editing types, ADAR-mediated adenine-to-inosine (A-to-I) editing is the predominant form in the brain. Accumulating evidence suggests that RNA editing patterns undergo significant alterations in PD patients. This review synthesizes current evidence within a three-layer framework: (1) evidence for RNA editing dysregulation in PD, emphasizing tissue-specific and context-dependent patterns; (2) downstream mechanistic pathways stratified by evidence strength; and (3) experimental models, translational applications, and limitations. A distinction between what is known versus what remains speculative is emphasized throughout. RNA editing changes in PD appear heterogeneous and context-dependent, with brain and peripheral blood showing distinct patterns. Whether editing changes represent disease drivers, compensatory responses, or downstream phenomena remains largely unresolved.

## 1 Introduction

PD is the second most common neurodegenerative disorder. Current dopamine replacement therapies can temporarily alleviate motor symptoms but fail to halt disease progression (Popescu et al., 2024; Lin et al., 2025; Ma et al., 2025; Hoseinipalangi et al., 2023; Conn and Jankovic, 2024; Jankovic and Tan, 2020). The pathogenesis of PD remains incompletely elucidated, with prevailing view implicating genetic susceptibility, environmental factors, and aging. Classic mechanisms include α-synuclein aggregation, mitochondrial dysfunction, neurotransmitter abnormalities, and neuroinflammation (Aoyama, 2021; Ye et al., 2023; Hattori and Sato, 2024; Kalyanaraman et al., 2024; Hong et al., 2024; Cheng et al., 2025; Belur et al., 2024).

RNA editing within the broader landscape of post-transcriptional regulation in PD. PD pathogenesis is increasingly discussed within a wider framework of epigenetic and post-transcriptional regulation. Beyond RNA editing, other RNA-based mechanisms including alternative splicing, non-coding RNAs, and RNA-binding protein dysfunction have been implicated in PD (Kim et al., 2007; Paccosi and Proietti-De-Santis, 2023). For example, alternative splicing of SNCA and MAPT generates isoforms with differential aggregation propensities (Miñones-Moyano et al., 2011), while dysregulation of microRNAs and long non-coding RNAs may affect α-synuclein expression and neuroinflammation (McMillan et al., 2017). Within this broader context, RNA editing—particularly A-to-I editing by ADAR enzymes—offers a mechanism for post-transcriptional sequence modification without genomic alteration, enabling dynamic regulation of gene function. This review focuses on RNA editing while acknowledging its place within a larger epitranscriptomic network relevant to PD.

RNA editing, as a post-transcriptional modification mechanism that does not alter genomic sequences but can precisely modulate gene expression and protein function, has been implicated in the development of the central nervous system, synaptic plasticity, and neuroimmune regulation in recent years (Conn and Jankovic, 2024; Jankovic and Tan, 2020). In PD, accumulating evidence suggests that alterations in RNA editing patterns may be involved in core pathological processes such as mitochondrial dysfunction, abnormal neurotransmitter signaling, and neuroinflammation, raising the possibility that RNA editing may represent a potential contributor to disease pathogenesis, while also offering novel avenues for diagnostic and therapeutic exploration.

However, despite initial progress in RNA editing research for PD, several critical unresolved questions remain in the field: First, the dynamic changes of RNA editing across different PD cell types and disease stages are not yet fully understood; Second, the causal relationships linking editing abnormalities to core pathologies (such as α-synuclein aggregation and mitochondrial autophagy dysfunction) remain to be established; Third, existing studies predominantly focus on A-to-I editing, while the potential roles of C-to-U editing and other non-coding RNA editing events in PD have received comparatively limited attention; Fourth, translating RNA editing findings into clinically applicable biomarkers or targeted therapeutic strategies faces considerable challenges related to delivery, specificity, and safety.

**Figure 1:**
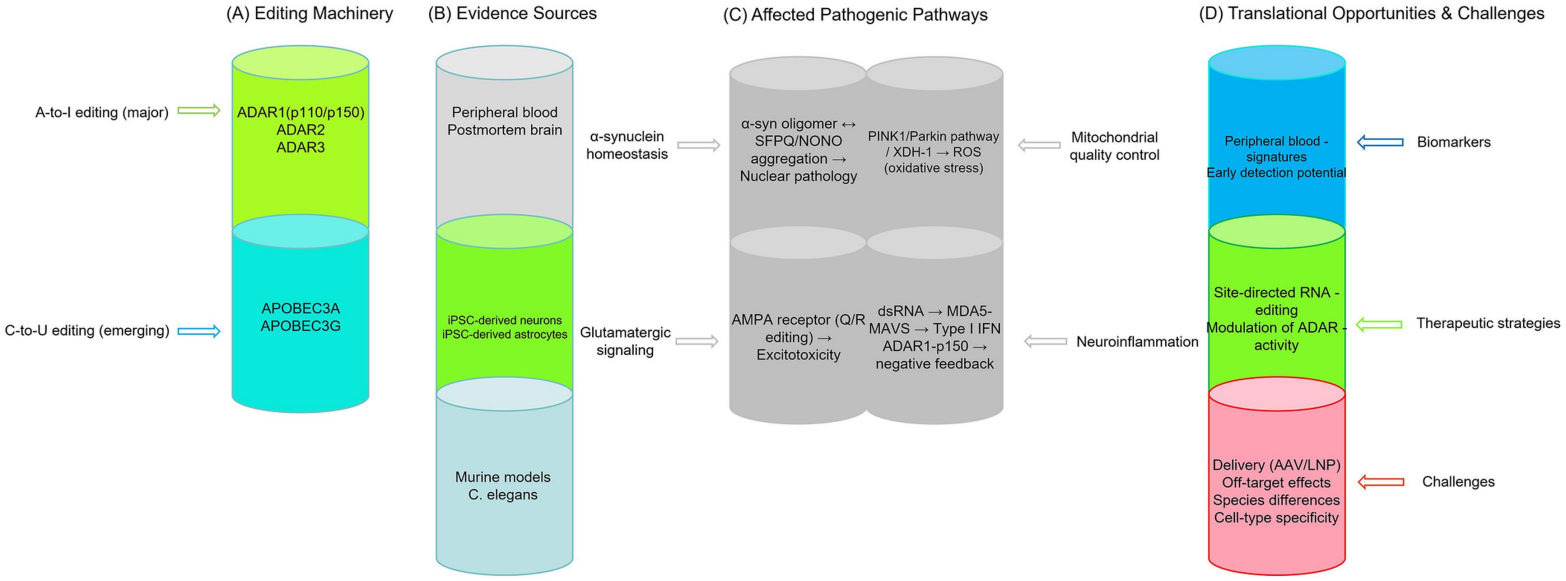
RNA editing in Parkinson’s disease: from dysregulation to pathogenesis and therapeutic opportunities. **(A)** Editing machinery. A-to-I editing is primarily mediated by ADAR1, ADAR2, and the catalytically inactive ADAR3. Emerging evidence implicates APOBEC family members (APOBEC3A, APOBEC3G) in C-to-U editing under inflammatory conditions. **(B)** Evidence sources. RNA editing alterations have been documented in postmortem PD brain tissues and peripheral blood samples. Human iPSC-derived neurons and astrocytes provide platforms for mechanistic dissection, while animal models (murine and *C. elegans*) offer insights into *in vivo* functions and species-conserved pathways. **(C)** Affected pathogenic pathways. RNA editing dysregulation has been linked to four PD-relevant pathways: (1) α-synuclein homeostasis, where α-syn oligomers may interact with SFPQ/NONO and nuclear RNA metabolism; (2) mitochondrial quality control, including potential effects on PINK1/Parkin-mediated mitophagy and oxidative stress (XDH-1/ROS); (3) glutamatergic signaling, particularly AMPA receptor Q/R site editing and excitotoxicity; and (4) neuroinflammation, involving ADAR1-mediated self-dsRNA editing and negative feedback regulation in astrocytes. **(D)** Translational opportunities and challenges. RNA editing signatures in peripheral blood are being explored as candidate non-invasive diagnostic biomarkers, though brain-periphery correlation requires rigorous validation. Therapeutic strategies under investigation include site-directed RNA editing to correct pathogenic mutations and modulation of endogenous editing enzyme activity. Key challenges for clinical translation include efficient and cell-type-specific delivery, off-target effects, species differences limiting preclinical models, and the need for precise spatiotemporal control of editing activity.

This review first systematically describes the molecular mechanisms underlying A-to-I and C-to-U RNA editing associated with the nervous system. Subsequently, drawing on peripheral samples, postmortem brain tissue samples, and disease-related cellular models and animal models, it examines current findings regarding RNA editing dysregulation in PD. Next, it analyzes how RNA editing alterations may influence key pathological pathways in PD, including α-synuclein pathology, mitochondrial quality control, neurotransmitter signaling, and neuroinflammatory responses. Finally, it discusses the translational potential of RNA editing-based biomarkers and therapeutic strategies, as well as current research limitations. By synthesizing key findings and unresolved questions in the field, this study aims to provide a systematic perspective on understanding the mechanisms of RNA editing in PD. The following schematic illustrates the current understanding (see Figure 1).

## 2 Section 1: evidence for RNA editing dysregulation in PD—a context-dependent landscape

RNA editing changes in PD appear not uniform but rather heterogeneous and context-dependent, varying by tissue type, genomic location, and cell type.

### 2.1 Molecular basis of RNA editing: ARADs, genomic landscape, and immunological function

#### 2.1.1 The ADAR family

Two types of RNA editing events currently under extensive investigation are: adenosine-to-inosine (A-to-I) editing mediated by the ADAR family, and cytosine-to-uridine (C-to-U) editing mediated by the APOBEC family (Tang et al., 2012; Savva et al., 2012; Smith et al., 2012; Sharma and Baysal, 2017). Among these, A-to-I editing is most prevalent in the brain and is thought to contribute to the maintenance of normal neurotransmitter receptors and ion channel function (Hwang et al., 2016; Nishikura, 2016).

The ADAR family comprises three members: ADAR1, ADAR2, and ADAR3. ADAR1 exhibits two subtypes: p110 (constitutively expressed in the nucleus) and p150 (cytoplasmic interferon-induced), with the core distinction lying in the p150-specific Zα domain. This domain has been implicated in the recognition of endogenous double-stranded RNA and inhibiting the type I interferon pathway, and its dysfunction has been associated with neuroinflammation (Langeberg et al., 2023; Chiang et al., 2021; Wang et al., 2014). ADAR2 is highly expressed in the brain and is responsible for site-specific precise editing, with substrates including key glutamate receptors such as GluA2 (Tamizkar and Jantsch, 2025; Cox et al., 2017; Christofi and Zaravinos, 2019). ADAR3 is specifically expressed in the central nervous system (CNS) and, although lacking catalytic activity, has been proposed to function as a negative regulator (Mladenova et al., 2018; Wang et al., 2019). In contrast, APOBEC family-mediated C-to-U editing in the nervous system remains under investigation, but it can be activated in inflammatory environments, leading to extensive nonsynonymous mutations in genes, suggesting a potential involvement in inflammation-related pathological processes of PD (Sharma et al., 2015; Sharma et al., 2017; Asaoka et al., 2019). Table 1 summarizes the major types of RNA editing and their characteristics in the nervous system.

**Table 1:** Major RNA editing types and their characteristics in the nervous system.

Editing type	Key enzyme	Hypotype	Organization/cell distribution and regulation	Known functions in the nervous system	Relevant evidence in PD
A-to-I	ADAR1	p110	Nucleus, constitutively expressed	Involved in RNA splicing and stability regulation	Expression levels reported to be reduced in brain tissue and peripheral blood of PD patients (Huang et al., 2025); may regulate astrocyte inflammation (D'Sa et al., 2025)
		p150	Cytoplasm, interferon-induced type	Key immune regulator that recognizes endogenous dsRNA and inhibits the type I interferon pathway	Mutations in the Zα domain have been associated with neurodegenerative diseases (Langeberg et al., 2023; Chiang et al., 2021; Wang et al., 2014); they exhibit feedback-mediated upregulation observed in PD may limit inflammation (D'Sa et al., 2025)
	ADAR2		Highly expressed in the brain	Responsible for site-specific editing (e.g., GluA2 Q/R site), important for neuronal survival	Abnormal activity has been linked to excitotoxicity; it can serve as a tool enzyme for targeted repair (e.g., PINK1) (Cox et al., 2017; Huang et al., 2025)
	ADAR3		Specifically expressed in the central nervous system, without catalytic activity	Proposed negative regulation of A-to-I editing	Significant downregulation in expression reported in iPS neurons from PD patients, accompanied by elevated global editing levels (Belur et al., 2024)
C-to-U	APOBEC family	APOBEC3A, APOBEC3G, etc.	Upregulated under stress conditions such as inflammation	Functional studies remain limited; may generate functionally variant proteins	May be activated in inflammatory environments and could potentially participate in pathological processes associated with PD neuroinflammation (Sharma et al., 2015; Sharma et al., 2017; Asaoka et al., 2019)

#### 2.1.2 Landscape of editing changes

A-to-I editing predominantly occurs in non-coding regions, with over 95% of editing events localized within Alu repeat elements (Lo Giudice et al., 2020; Breen et al., 2019). Analysis of genomic distribution of editing events in PD patients revealed that the majority of events were located in protein-coding genes (93.49%), non-exon/splicing regions (99.77%), and Alu repeat sequences (91.76%). These findings align with previously reported RNA editing site preferences and help delineate the overall landscape of RNA editing alterations in PD (Wu et al., 2021; Chigaev et al., 2019). This distribution pattern suggests that A-to-I editing may primarily function through post-transcriptional regulatory processes such as RNA splicing, stability, and circRNA generation, with potential roles in neurodevelopment, ion channel function, and neuronal signaling.

#### 2.1.3 ADAR1’s classical immunological function

ADAR1 is thought to contribute to immune tolerance of endogenous nucleic acids through its editing activity. The core mechanism involves ADAR1 editing adenine (A) in endogenous double-stranded RNA (dsRNA) to inosine (I), thereby marking it to prevent recognition by innate immune sensors (Hu et al., 2023; Liddicoat et al., 2015; Mannion et al., 2014). When ADAR1 is dysfunctional or downregulated, unedited dsRNA (particularly originating from transposable elements such as Alu and LINE-1) accumulates extensively, leading to abnormal activation of the MDA5-MAVS signaling pathway. This results in robust expression of type I interferon (IFN) and interferon-stimulated genes (ISGs), driving sterile inflammatory responses (Hu et al., 2023). Additionally, loss of ADAR1 function causes accumulation of Z-RNA and activation of ZBP1, triggering apoptosis and necroptosis, which further exacerbates tissue damage (de Reuver and Maelfait, 2024; Karki and Kanneganti, 2023; Wang et al., 2024; Liu et al., 2024). The aforementioned immunological principles are equally applicable to the central nervous system and have been validated in the functional regulation of glial cells.

### 2.2 Tissue-specific patterns: brain versus peripheral blood

#### 2.2.1 Brain tissue evidence

RNA sequencing analysis of autopsy samples from the prefrontal cortex of 29 PD patients and 44 healthy individuals demonstrated universally reduced editing levels in protein-coding gene exons among PD patients, accompanied by significant downregulation of ADAR1 and ADARB1 gene expression (Huang et al., 2025). This pattern has been reported across multiple brain region studies.

#### 2.2.2 Peripheral blood evidence

Peripheral blood data show greater variability. Wu et al. analyzed whole blood RNA sequencing data from 372 PD patients, revealing 9,897 A-to-I editing events associated with 6,286 genes. Over 26% of these events were significantly correlated with the onset and progression of PD, with 1,224 PD-specific RNA editing events identified (Wu et al., 2023). Data from PPMI consortium further validated the markedly lower overall A-to-I editing frequency in peripheral blood of PD patients (Parkinson Progression Marker Initiative, 2011; Marek et al., 2018; Pozdyshev et al., 2021). Unlike brain tissue, ADARB1 shows mild upregulation in peripheral blood (Song et al., 2023), suggesting differences between CNS and peripheral compartments that warrant further investigation.

### 2.3 Cell-type specificity: resolving apparently contradictory findings

Bulk tissue analysis shows decreased global editing (Huang et al., 2025). However, Belur et al. used iPSC-derived dopaminergic neurons from PD patients and found ADAR3 downregulation (75%) associated with elevated global A-to-I editing (Belur et al., 2024).

These findings may be reconciled by considering: (1) bulk tissue includes multiple cell types; (2) opposing directional changes may occur in different cell types; (3) ADAR3 may act as a negative regulator whose downregulation disinhibits editing.

### 2.4 Genetic evidence for causal associations (preliminary)

Li et al. (2024) identified 17 A-to-I editing sites with potential causal associations to PD pathogenesis through integration of Mendelian randomization analysis. These sites were located within known PD risk genes NCOR1, KANSL1, and BST1, among which 6 sites were associated with increased PD risk while the remaining 11 exhibited potential protective effects (Li et al., 2024). This finding provides genetic clues regarding possible causal relationships between editing and PD. However, whether these sites directly mediate the function of risk genes and whether their effects are attributable to editing itself or linked genetic variants still require functional experimental validation.

### 2.5 Summary of section 1

Table 2 shows context-dependent evidence summary.

**Table 2:** Context-dependent evidence summary.

Context	Key finding	Evidence status
Brain tissue (bulk)	Decreased exonic editing, reduced ADAR1/ADARB1	Replicated
Peripheral blood	Reduced global editing; ADARB1 may be upregulated	Preliminary
iPSC dopaminergic neurons	Increased editing (via ADAR3 downregulation)	Preliminary (single study)
Causal associations (MR)	17 sites in PD risk genes	Preliminary (needs validation)

## 3 Section 2: downstream mechanistic pathways—stratified by evidence strength

This section organizes proposed mechanisms into three tiers based on evidence strength (summarized in Table 3). Strong evidence pathways have been validated in human cells/tissues with causal manipulations; moderate evidence pathways show consistent associations but lack definitive causality in relevant cell types; speculative pathways are biologically plausible but lack direct PD-specific evidence.

**Table 3:** Evidence strength assessment of RNA editing-related mechanisms in PD.

Mechanistic pathway	Evidence strength	Key supporting evidence	Key gaps
RNA-binding protein dysfunction (SFPQ/NONO)	Strong	Human iPSC neurons, PD brain tissue, ADAR mutant rescue	Spatiotemporal distribution across Braak stages
Neuroinflammation (ADAR1-dsRNA-MDA5)	Strong	Human astrocytes, α-syn oligomer direct activation, feedback loop validated	Causal direction (driver vs. compensatory)
Mitochondrial/oxidative stress (XDH-1/ROS)	Moderate	*C. elegans* model, conserved pathway	Mammalian validation lacking
Glutamate receptor editing (GluA2 Q/R)	Moderate	PD brain editing changes, ALS precedent	Causality not established in SNc DA neurons
5-HT receptor editing & non-motor symptoms	Speculative	Plausible from other diseases	No direct PD evidence
PINK1/Parkin direct editing regulation	Speculative	Engineered editing repair only	No endogenous evidence

### 3.1 Strong evidence pathways

#### 3.1.1 RNA editing and α-synuclein pathology: RNA-binding protein dysfunction

α-synuclein, encoded by the SNCA gene, is the core pathogenic protein in PD. Currently, there is no definitive evidence indicating whether its encoded transcript itself serves as a direct editing target of the ADAR family (Starr et al., 2023). However, recent studies have revealed potential indirect interactions between α-synuclein pathology and RNA metabolic regulatory networks.

##### 3.1.1.1 Established associations

Multiple independent studies suggest that abnormal aggregation of α-synuclein may impair the function of RNA-associated proteins. In neurons and brain tissues of PD patients, α-synuclein has been observed to exhibit co-localization with RNA-binding proteins SFPQ and NONO within the nucleus (Belur et al., 2024). *In vitro* experiments further demonstrate that different aggregation forms of *α*-synuclein exhibit distinct biological effects: soluble α-synuclein oligomers significantly influence the aggregation state of SFPQ, inducing the formation of high-molecular-weight species, while SFPQ conversely promotes the transformation of α-synuclein oligomers into precipitated aggregates, suggesting a mutually reinforcing synergistic aggregation relationship between the two (Belur et al., 2024). Concurrently, multiple studies based on PD patient brain tissues and iPSC neurons have reported alterations in global RNA editing patterns, including abnormal expression levels of ADAR enzymes and changes at specific editing sites (Belur et al., 2024; Huang et al., 2025; Li et al., 2024).

Beyond the SFPQ/NONO axis, α-synuclein has been shown to interact more broadly with the RNA metabolism machinery. Hallacli et al. (2022) demonstrated that α-synuclein physically interacts with processing body (P-body) components—cytoplasmic membraneless organelles specialized in mRNA degradation and storage. Notably, α-synuclein was shown to modulate mRNA stability through this interaction, affecting the turnover rates of target transcripts. In α-synuclein-expressing Drosophila models, brain-specific knockdown of XRN1, a P-body-associated 5′ → 3′ ribonuclease, exacerbated locomotor deficits, indicating that P-body genes can modulate α-synuclein-mediated toxicity (Kapetanou et al., 2022). Given that ADAR enzymes access their substrates within dynamic RNA structural contexts, *α*-synuclein-induced perturbations of global RNA metabolism—including altered mRNA stability and sequestration of RNA species into P-bodies—could indirectly influence the landscape of ADAR-mediated editing.

The pathogenic impact of α-synuclein accumulation extends further to broader disruption of neuronal proteostasis. Studies using patient-derived midbrain cultures that develop α-synuclein pathology through endogenous expression of PD-causing mutations have revealed that proteostasis disruption occurs at multiple levels: synthesis and folding in the endoplasmic reticulum (ER), ER-Golgi trafficking, and autophagic-lysosomal clearance (Paccosi et al., 2020). Of particular relevance to RNA editing is the observation that this proteostatic collapse involves numerous RNA-binding proteins and splicing factors whose functional integrity depends on proper protein folding and trafficking. As ADAR enzymes themselves are subject to proteostatic regulation, α-synuclein-induced ER stress and autophagic dysfunction could secondarily impair ADAR protein stability or activity. This raises the possibility that α-synuclein pathology acts through a dual mechanism: directly disrupting RNA-binding protein function (SFPQ/NONO, P-body components) while also compromising the proteostatic environment required for ADAR enzyme function and RNA substrate availability.

##### 3.1.1.2 Proposed causal cascade and current limitations

Based on the aforementioned correlations, a causal cascade has been proposed: SNCA pathology (upstream driver; encompassing both SNCA multiplication mutations in familial forms and α-synuclein aggregation in sporadic contexts) → dysfunction of RNA-binding proteins SFPQ/NONO → nuclear retention of key transcripts, particularly those involved in axonal, synaptic, and mitochondrial function → secondary alterations in ADAR-mediated RNA editing (Belur et al., 2024; Starr et al., 2023). This cascade is supported by data from patient-derived iPSC-derived dopaminergic neurons—the cell type most relevant to PD pathology—where these molecular events have been experimentally linked. Notably, the available experimental evidence spans a range of genetic and disease contexts: the initial findings in iPSC neurons from a SNCA triplication line (familial PD) have been complemented by observations in sporadic dementia with Lewy bodies (DLB) postmortem brain tissue, providing a degree of genetic and disease heterogeneity that includes both familial and sporadic α-synucleinopathies (Belur et al., 2024).

A key mechanistic link bridging SFPQ/NONO aggregation and downstream RNA editing changes may involve ADAR3. In the same SNCA triplication iPSC-derived neurons, Belur et al. (2024) observed a marked (~75%) downregulation of ADAR3 expression, which occurred in the context of SFPQ/NONO dysfunction and nuclear RNA retention. ADAR3, a catalytically inactive member of the ADAR family that is proposed to function as a negative regulator of A-to-I editing, is specifically expressed in the central nervous system. Its downregulation would be expected to disinhibit ADAR1- and ADAR2-mediated editing, consistent with the observed global elevation of A-to-I editing levels in these cells. Thus, SFPQ/NONO aggregation → ADAR3 transcriptional or post-transcriptional downregulation → disinhibition of ADAR1/ADAR2 → elevated global editing represents a plausible molecular subcascade that connects nuclear RNA-binding protein pathology to altered RNA editing output. Whether ADAR3 downregulation is a direct consequence of SFPQ/NONO dysfunction (e.g., due to nuclear retention of ADAR3 transcripts or impaired splicing regulation) or occurs through a parallel pathway remains to be determined.

However, despite this evidence of a directional cascade and the inclusion of both familial and sporadic models, several important gaps remain. First, generalizability across broader genetic backgrounds and cell types: while the current data cover SNCA triplication and sporadic DLB contexts, it remains unknown whether the same cascade operates in patients with other SNCA mutations (e.g., A53T, E46K, A30P) or in idiopathic PD without DLB pathology. Similarly, whether this mechanism is active in vulnerable cell populations beyond iPSC-derived dopaminergic neurons (e.g., substantia nigra dopamine neurons *in situ*, or other affected brain regions such as the locus coeruleus) requires independent validation. Second, spatiotemporal distribution: The incidence and progression of α-synuclein/SFPQ co-aggregation across Braak stages and brain regions (e.g., substantia nigra compacta versus cortex) remain to be characterized. Third, causality at the organismal level: While the *in vitro* data support a directional cascade, definitive proof that SFPQ/NONO dysfunction is necessary and sufficient for PD-relevant neurodegeneration *in vivo* is still lacking. Fourth, completeness of the cascade: The precise molecular steps from SFPQ aggregation to ADAR3 downregulation and subsequent changes at specific editing sites have not been fully delineated.

In summary, the interaction between α-synuclein pathology and the RNA editing system represents an intriguing yet nascent research direction in the study of PD pathogenesis. Current evidence supports the existence of a functional association between the two, but it remains premature to generalize these preliminary observations into a universal mechanism of RNA editing alterations in PD or to establish it as a definitive pathogenic pathway. Future systematic validation across independent cohorts, diverse models, and different disease stages will be required to clarify the true contribution of this pathway.

#### 3.1.2 RNA editing and neuroinflammation: ADAR1-mediated negative feedback

Neuroinflammation is thought to act as an important contributor to the pathological progression of PD, with ADAR1-mediated RNA editing implicated in this process.

##### 3.1.2.1 Translational significance in the central nervous system: inflammatory regulation by glial cells

In neurodegenerative diseases such as Alzheimer’s disease (AD), downregulation of ADAR1 expression has been observed in the brain tissue of AD patients, accompanied by inflammatory activation (Chung et al., 2018; McEntee et al., 2023). McEntee et al. (2023) demonstrated in human astrocytes that inhibition of ADAR1 leads to activation of the interferon signaling pathway and accumulation of transposable element transcripts. Feedback-mediated upregulation of ADAR1 in astrocytes may suppress immune responses triggered by cytoplasmic dsRNA by editing endogenous dsRNA (Goldeck et al., 2022; Cottrell et al., 2024; Zhou et al., 2019; Scadden, 2007).

Upstream of ADAR1 dysfunction, emerging evidence points to epigenetic destabilization as a potential driver of dsRNA accumulation in PD. Using single-nucleus multi-omics, Sun et al. identified trans-disease epigenetic signatures in postmortem brains of multiple neurodegenerative disorders, including PD, characterized by loss of heterochromatin marks (H3K9me3 and H3K27me3) and consequent derepression of repetitive elements such as LINE-1 (Butto et al., 2024). LINE-1 transcripts form double-stranded RNA structures that are endogenous substrates for ADAR1. When ADAR1 editing capacity is overwhelmed or when epigenetic derepression exceeds a critical threshold, unedited dsRNA accumulates and triggers MDA5-MAVS-mediated type I interferon responses—a mechanism established in autoimmune disorders but increasingly recognized in neurodegenerative contexts. This epigenetic-dsRNA-ADAR1 axis may therefore represent an upstream pathogenic cascade independent of, or synergistic with, α-synuclein pathology.

The study by D'Sa et al. (2025) further suggests that pathological α-synuclein oligomers may serve as endogenous danger signals under PD pathology, recognized by pattern recognition receptors of astrocytes (e.g., TLR4, TLR3, RIG-I), thereby directly initiating neuroinflammation. This recognition triggers a robust antiviral-like response characterized by activation of the type I interferon signaling pathway and substantial secretion of pro-inflammatory cytokines (e.g., TNF-α, IL-6, IL-1β). Notably, the study also revealed that during inflammatory responses, the type I interferon pathway not only promotes inflammatory cytokine release but also induces upregulation of ADAR1 gene expression and its subtype transition from p110 to p150 (D'Sa et al., 2025). Cytoplasmic ADAR1-p150 appears to exert negative feedback inhibition on immune responses by catalyzing A-to-I editing of endogenous dsRNA.

##### 3.1.2.2 PD-specific evidence—direct association with *α*-synuclein pathology

Evidence directly linking the aforementioned mechanisms to the core pathology of PD is gradually accumulating.

Firstly, α-synuclein oligomers themselves have been shown to act as danger signals capable of triggering inflammatory responses in astrocytes. Studies by D'Sa et al. (2025) indicate that pathological α-synuclein oligomers are sufficient to directly activate the aforementioned ADAR1-associated inflammatory feedback loop. This provides a plausible molecular explanation for one potential initiation mechanism of neuroinflammation in PD.

Secondly, aberrations in ADAR-related pathways have been documented in both brain tissues of PD patients and iPSC-derived models. Although studies directly measuring ADAR1 expression levels in PD brains have yielded inconsistent results, alterations in global RNA editing patterns have been confirmed in multiple studies (Belur et al., 2024; Huang et al., 2025; Li et al., 2024). These changes may indirectly modulate the intensity of inflammatory responses by influencing the editing status of dsRNA.

Furthermore, the study by Belur et al. revealed a non-canonical pathogenic mechanism: in iPSC-derived neurons from PD patients, ADAR3 expression was significantly downregulated, leading to abnormally elevated global A-to-I editing levels (Belur et al., 2024). Artificial overexpression of hyperactive ADAR1 or ADAR2 mutants in healthy neurons was sufficient to directly induce the formation of nuclear pathological inclusion bodies (Belur et al., 2024; Wang et al., 2015). This finding raises the possibility that abnormal elevation of A-to-I editing levels within neurons could itself constitute a pathogenic factor, potentially driving neuropathological processes through mechanisms independent of their immunomodulatory functions.

Current evidence suggests that ADAR1-mediated RNA editing may contribute to the regulation of neuroinflammation during PD, but whether it functions primarily as a driver, limiter, or bystander of the disease remains to be further elucidated. Clarifying this core issue will be pivotal for advancing this field from mechanistic research toward intervention applications.

### 3.2 Moderate evidence pathways

#### 3.2.1 RNA editing and mitochondrial quality control

The mitochondrial quality control pathway, particularly PINK1/Parkin-mediated autophagy, is thought to play an important role in the pathogenesis of PD (Prasuhn and Brüggemann, 2021; Liao et al., 2017). Defects in nuclear genes such as PRKN and PINK1 disrupt the function of electron transport chain complexes, leading to reduced ATP synthesis and excessive production of reactive oxygen species (ROS), which induce oxidative damage to lipids, proteins, and DNA (Masaldan et al., 2022). When mitochondria are damaged, PINK1 stabilizes on their outer membrane and phosphorylates Parkin and mitochondria-associated proteins, triggering the translocation of Parkin from the cytoplasm to damaged mitochondria, thereby initiating the autophagy process (Masaldan et al., 2022). However, PD-associated loss-of-function mutations in PINK1 (e.g., the nonsense mutation W437X) severely impair PINK1’s ability to recruit Parkin, resulting in abnormal accumulation of dysfunctional mitochondria and ultimately neuronal damage (Masaldan et al., 2022).

#### 3.2.2 RNA editing in glutamatergic signaling and excitotoxicity

Glutamate-mediated excitotoxicity has been proposed as one of the key mechanisms underlying dopaminergic neuron loss in PD. The precise functional regulation of ionotropic glutamate receptors, particularly AMPA receptors, relies on ADAR2-mediated RNA editing.

RNA sequencing analysis of autopsy samples from the prefrontal cortex of PD patients revealed multiple editing sites on the mRNA of ionotropic glutamate receptors (including kainate receptors and AMPA receptor subunits), with altered editing levels observed in PD (Huang et al., 2025). Pozdyshev et al. (2021) proposed that these changes may represent compensatory responses of the nervous system to maintain homeostasis under pathological stress in PD.

Dysregulation of GluA2 Q/R site editing is not PD-specific. In amyotrophic lateral sclerosis (ALS), downregulation of ADAR2 and insufficient GluA2 Q/R site editing have been shown to be core mechanisms underlying the selective death of spinal motor neurons (Hideyama et al., 2012). This similarity raises the possibility that excitatory toxicity caused by RNA editing defects could represent a common pathogenic pathway in multiple neurodegenerative diseases. However, different neuronal populations exhibit selective vulnerability to specific editing defects: spinal motor neurons are affected in ALS, whereas dopaminergic neurons in the substantia nigra are hypothesized to be key targets of such editing defects in PD (Huang et al., 2025).

Substantia nigra dopaminergic neurons exhibit unique susceptibility to excitotoxicity, which has been associated with their specialized calcium buffering capacity, pacing activity patterns, and glutamate receptor subunit composition. It has been hypothesized that reduced editing levels at the AMPA receptor Q/R sites could enhance receptor permeability to calcium ions, thereby increasing neuronal vulnerability to excitotoxic damage. Although this mechanism has been relatively well-established in ALS, a direct causal relationship between editing abnormalities and neuronal death remains to be established in PD substantia nigra neurons.

#### 3.2.3 Transcriptional stress and p53-mediated vulnerability: a unifying mechanism

The convergence of multiple RNA-related pathologies—ADAR1 dysfunction, nuclear RNA retention, and global editing downregulation—points to a common downstream consequence: impaired global transcription. Nixon et al. demonstrated that activated p53 can directly block the elongation phase of mRNA synthesis through physical interaction with the TFIIH transcription complex, leading to global transcriptional arrest that precedes and predicts neuronal death (Proietti De Santis et al., 2001). This p53-dependent transcription termination mechanism is particularly relevant to PD for three reasons.

First, multiple PD-relevant stressors converge on p53 activation. Both α-synuclein pathology and dsRNA-mediated innate immune signaling have been shown to activate p53 pathways. Second, dopaminergic neurons exhibit unique vulnerabilities to transcriptional stress. Their long, highly arborized axons require continuous supply of newly synthesized proteins (e.g., mitochondrial components, synaptic vesicle proteins), a demand that cannot be met under conditions of global transcription inhibition. Third, SFPQ/NONO dysfunction—documented in PD iPSC neurons—impedes nuclear export of key transcripts, creating a functional state equivalent to “transcription scarcity” at the cytoplasm even if transcription per se is intact.

These observations suggest a unifying framework: RNA editing dysregulation, whether through ADAR loss-of-function, epigenetic dsRNA overload, or SFPQ/NONO-mediated RNA retention, ultimately imposes transcriptional or transcript-availability stress on neurons. Dopaminergic neurons, with their high metabolic and protein turnover demands, may be exquisitely sensitive to such stress. This framework also explains why bulk tissue analyses showing modest global editing changes could still have clinically significant consequences in vulnerable neuronal populations.

Importantly, this transcriptional stress pathway operates in parallel with, and may synergize with, the proteostatic disruptions described in Section 3.1.1. While α-synuclein pathology impairs protein folding and clearance, transcriptional stress reduces the synthesis of new proteins—including those needed to maintain proteostasis. The convergence of these two insults may create a vicious cycle that drives progressive neuronal dysfunction.

### 3.3 Speculative pathways

#### 3.3.1 Serotonin receptor editing and non-motor symptoms

Non-motor symptoms (such as depression and anxiety) are prevalent in patients with PD, significantly impairing quality of life, yet their molecular mechanisms remain incompletely understood. RNA editing regulation of serotoninergic receptors offers a novel perspective for understanding these symptoms.

The serotonin 2C receptor (5-HT2C receptor) and serotonin 2B receptor (5-HT2B receptor) are known hotspots for A-to-I editing. ADAR1 has been implicated in the editing of 5-HT2C receptor precursor mRNA, and variations in editing levels can alter the receptor’s G protein-coupling efficiency and pharmacological properties (Burns et al., 1997; Dorrity et al., 2023). Given the central role of the serotonin system in mood regulation, aberrations in receptor editing may be relevant to non-motor symptoms such as depression and anxiety in patients with PD (Cheng et al., 2025). Although this hypothesis is biologically plausible, direct evidence linking abnormal 5-HT receptor editing to non-motor symptoms in PD remains limited. Current understanding primarily relies on indirect evidence from other diseases or models, such as ADAR2-mediated 5-HT2B receptor editing leading to functional loss in hyperammonemia models (Yue et al., 2019). No studies have directly compared 5-HT receptor editing levels in PD patients with those related to severity of non-motor symptoms. Additionally, the occurrence of non-motor symptoms involves multiple brain regions and diverse neurotransmitter systems, making it methodologically challenging to directly correlate specific receptor editing alterations with complex behavioral phenotypes.

#### 3.3.2 Direct PINK1/Parkin editing regulation

While engineered RNA editing can therapeutically correct PINK1 mutations (Cox et al., 2017; Li et al., 2025), there is currently no evidence that endogenous RNA editing directly regulates PINK1 or Parkin transcripts under physiological or pathological conditions. This remains a speculative possibility requiring future investigation.

## 4 Section 3: experimental models, translational applications, and synthesis

### 4.1 Experimental models and their limitations

#### 4.1.1 Human iPSC-derived neuronal and glial models

Human-derived iPSC models enable the simulation of human disease processes in controlled environments, providing a critical tool for investigating the causal effects of RNA editing. In patient-derived iPSC-derived dopaminergic neurons—the cell population most directly relevant to PD pathology—Belur et al. (2024) observed a significant downregulation of ADAR3 expression (approximately 75%), leading to elevated global A-to-I editing levels. This was accompanied by dysfunction of SFPQ/NONO RNA-binding proteins and nuclear retention of key transcripts involved in axonal, synaptic, and mitochondrial function, providing a mechanistic link from SNCA mutations to downstream RNA processing abnormalities and, ultimately, to neuronal vulnerability. Notably, overexpression of hyperactive ADAR1 or ADAR2 mutants in healthy iPSC neurons was sufficient to directly induce similar pathological changes, demonstrating that A-to-I editing abnormalities can act as pathogenic drivers in this experimental context (Belur et al., 2024; Wang et al., 2015). Astrocyte models revealed the bidirectional regulatory role of RNA editing in neuroinflammation. D'Sa et al. (2025) found that pathological α-synuclein oligomers are recognized by astrocytes, triggering an interferon type I response accompanied by feedback-upregulated ADAR1-p150 subtypes. ADAR1 is thought to prevent excessive activation of immune sensors by editing endogenous double-stranded RNA, thereby mitigating inflammation (Hu et al., 2023; Liddicoat et al., 2015; Mannion et al., 2014). Although causal links between editing abnormalities and core pathologies (protein aggregation, inflammation) have been established in human cell models and cell-type-specific functional differences have been identified, *in vitro* culture environments cannot fully simulate complex intracellular interactions in the brain, and most studies remain at the single-cell level.

#### 4.1.2 Animal models and their limitations

Mammalian models serve as indispensable tools for investigating RNA editing in the function of intact nervous systems. Yue et al. utilized a urease-induced high-ammonia mouse model to demonstrate that metabolic stress can upregulate ADAR2 activity, increase 5-HT2B receptor editing in astrocytes, and lead to receptor dysfunction, offering a potential molecular explanation for PD-related non-motor symptoms (Yue et al., 2019). However, rodent models exhibit significant limitations in simulating specific RNA editing-related pathologies. Belur et al. (2024) found that the cascade of SFPQ/NONO dysfunction, nuclear RNA retention, and RNA editing abnormalities observed in human iPSC-derived dopaminergic neurons was not replicated in transgenic mice expressing human A53T α-synuclein. This discrepancy—not a limitation of the human cell model but rather a reflection of true species differences—has been attributed to the absence of primate-specific genomic elements (e.g., reverse Alu elements and NEAT1_2 lncRNA) in the mouse genome, which precludes recapitulation of certain human-specific RNA processing pathways. In contrast, invertebrate models facilitate the identification of conserved regulatory networks. Starr et al. (2023) discovered in *Caenorhabditis elegans* that ADR-2 (an ADAR homolog) modulates α-synuclein toxicity by regulating the WHT-2 → XDH-1/ROS pathway, directly linking RNA editing to oxidative stress damage. Animal experiments have validated the pathological effects of specific editing events at the *in vivo* level and revealed conserved regulatory mechanisms across species. However, substantial interspecies differences (particularly the absence of Alu elements) limit the utility of mouse models in simulating certain human-specific RNA editing pathologies.

### 4.2 Translational opportunities and challenges

#### 4.2.1 Biomarker development

to-I editing alterations have been detected at the earliest stages of disease onset—even in the early Braak stage characterized by only brainstem Lewy bodies without cortical inclusion bodies, where specific transcript editing levels have been reported to show significant elevation (Belur et al., 2024). This feature suggests that RNA editing may reflect disease initiation events and holds theoretical potential as an early biomarker. Disease-associated editing events are stably detected in peripheral blood (Wu et al., 2023; Parkinson Progression Marker Initiative, 2011; Marek et al., 2018; Pozdyshev et al., 2021), providing a feasible foundation for developing non-invasive diagnostic tools.

However, translating peripheral blood RNA editing characteristics into reliable clinical biomarkers faces multiple unresolved core challenges: The association between peripheral changes and intracerebral pathology remains unclear—currently, it is uncertain to what extent detected RNA editing alterations in peripheral blood reflect intracerebral pathological states. Significant differences exist in RNA editing profiles between central and peripheral tissues—for instance, studies on brain tissue consistently report decreased ADAR editing levels, whereas ADARB1 exhibits mild upregulation at the RNA level in peripheral blood (Song et al., 2023). This inconsistency suggests that assumptions directly using peripheral blood editing profiles to infer intracerebral pathology may be overly simplistic. Peripheral blood editing changes may primarily reflect systemic inflammatory states or alterations in immune cell subset proportions rather than brain-specific pathology. Interference from multiple confounding factors: Peripheral blood RNA editing levels may be influenced by various non-disease-specific factors, including age, drug effects, systemic inflammation, and changes in blood cell composition. Insufficient longitudinal stability and reproducibility: Most current studies adopt cross-sectional designs, lacking longitudinal tracking data on editing changes across time points in the same patients. Questions regarding the reproducibility of candidate biomarkers across cohorts, their dynamic evolution during disease progression, and their ability to distinguish PD from other neurodegenerative disorders remain unanswered. Existing studies generally have small sample sizes and predominantly focus on European populations, with their generalizability yet to be validated (Song et al., 2023; Li et al., 2024). Technical standardization has not yet been established: There is a lack of unified standardized protocols for RNA editing detection, with variations observed in RNA extraction methods, sequencing platforms, and bioinformatics analysis workflows. This results in difficulties in directly comparing findings across different studies and increases the complexity of biomarker validation.

#### 4.2.2 Therapeutic intervention strategies

RNA editing technology has opened new avenues for precision therapy in PD. Currently, two primary strategic approaches are under investigation: first, utilizing engineered RNA editing tools to directly repair pathogenic gene mutations; second, restoring editing homeostasis by modulating the activity of endogenous editing enzymes.

In gene repair, RNA editing strategies targeting PINK1 mutations have achieved proof-of-concept. By designing specific guide RNAs that mimic the RNA structure of endogenous ADAR2 substrates, ADAR2 enzymes can be directed to precisely repair the W437X mutation site in the PINK1 gene, successfully reactivating mitochondrial autophagy in cell models (Cox et al., 2017; Li et al., 2025). A potential advantage of this strategy is that it acts exclusively on RNA transcripts without altering genomic DNA sequences, theoretically offering reversibility and a favorable safety profiles relative to DNA-editing approaches (Tao et al., 2023; Yin et al., 2017). In homeostatic regulation, given ADAR1’s implicated role in neuroinflammation, restoring its function or modulating subtype switching has been proposed as a potential therapeutic interventions for suppressing pathological inflammation (Cheng et al., 2025; D'Sa et al., 2025). Additionally, antisense oligonucleotide (ASO)-mediated site-specific editing regulation is currently under investigation (D'Sa et al., 2025; Wu et al., 2023).

A critical bottleneck for clinical translation is the lack of delivery systems that combine efficient blood–brain barrier (BBB) penetration, cell-type specificity, and acceptable safety profiles. Current options include viral vectors (AAV, lentivirus) and non-viral vectors (lipid nanoparticles, LNP). AAV offers natural neurotropism and low immunogenicity but has limited packaging capacity (<4.7 kb). Lentiviruses carry larger transgenes but raise insertional mutagenesis concerns (Booth et al., 2023; Han et al., 2023). LNP avoids genomic integration but achieves poor brain parenchyma delivery efficiency (<0.1%) (Li et al., 2025; Zhao et al., 2023; Mukai et al., 2022). No existing system enables non-invasive, precise targeting of substantia nigra dopaminergic neurons or specific glial subsets without off-target effects or immune activation.

It must be emphasized that most of the aforementioned therapeutic strategies are currently in the proof-of-concept stage at the cellular level. Each translational step—from cellular models to animal models and then to clinical applications—faces significant challenges regarding efficiency, specificity, and safety.

### 4.3 Synthesis: key findings and remaining uncertainties

#### 4.3.1 Summary of main findings

Based on the systematic review presented above, the main findings can be summarized as follows:

First, RNA editing appears to be extensively dysregulated in PD. Large-scale transcriptomic analyses from peripheral blood and postmortem brain tissues consistently demonstrate disease-specific A-to-I editing events in PD patients, with significantly reduced overall editing frequency (Huang et al., 2025; Wu et al., 2023; Parkinson Progression Marker Initiative, 2011; Marek et al., 2018; Pozdyshev et al., 2021).

Second, abnormal RNA editing has been associated with core pathological pathways implicated in PD. By integrating evidence from human iPSC models, animal models, and clinical samples, this review describes proposed mechanisms by which RNA editing may influence four key areas: the interaction between α-synuclein and RNA metabolism, regulation of mitochondrial autophagy and oxidative stress, glutamate receptor editing and excitatory toxicity, and ADAR1-mediated neuroinflammatory negative feedback regulation (Belur et al., 2024; Cox et al., 2017; Huang et al., 2025; D'Sa et al., 2025; Starr et al., 2023).

Third, RNA editing shows potential but also faces substantial challenges in diagnostic and therapeutic translation. Peripheral blood editing signatures are being evaluated as candidates for non-invasive biomarkers, while site-specific RNA editing tools offer a novel approach for repairing pathogenic mutations (Cox et al., 2017; Wu et al., 2023). However, issues such as delivery efficiency, off-target risks, species variability, and cell type specificity remain major bottlenecks in clinical translation (Belur et al., 2024; Li et al., 2025; Booth et al., 2023; Han et al., 2023; Zhao et al., 2023; Mukai et al., 2022).

Fourth, current evidence primarily establishes associations with causal mechanism remaining to be defined. For the vast majority of editing events, whether they represent disease drivers, compensatory responses, or downstream phenomena requires further elucidation.

#### 4.3.2 Remaining uncertainties

Despite progress, the following critical uncertainties remain:

Causal direction unknown. Does aberrant RNA editing drive PD pathology, or is it a consequence of neurodegeneration?

Cell-type specificity unresolved. Do RNA editing changes in neurons, astrocytes, and microglia exert convergent or opposing effects on disease progression?

Dynamic evolution uncharacterized. How do RNA editing profiles change across Braak stages and in different brain regions?

Brain-periphery relationship undefined. Can peripheral blood editing signatures reliably reflect intracerebral pathology?

Clinical validation absent. No RNA editing-based diagnostic or therapeutic strategy has yet undergone prospective clinical validation.

### 4.4 Future prospects

Based on the current research progress and limitations, future studies may benefit from focusing on following directions:

First, developing an experimental systems capable of establishing causal relationships. Future research will need to transition from “association description” to “causal validation.” This will likely involve conducting gain-of-function and loss-of-function experiments in cellular and animal models, utilizing antisense oligonucleotides or engineered ADAR systems to precisely regulate editing levels in specific cell types, and observing their effects on α-synuclein aggregation, mitochondrial function, neuroinflammation, and neuronal survival. Particular attention should be directed toward substantia nigra dopaminergic neurons—the most vulnerable cell population in PD—rather than relying on more readily available cortical tissues or mixed cell populations.

Second, generating dynamic cell type-specific editing maps. Emerging single-cell and spatial transcriptomics technologies could be employed to map the dynamic evolution of RNA editing profiles across different brain regions and cell types (dopaminergic neurons, astrocyte subtypes, microglia) during the progression of PD.

Third, addressing the bottleneck of human-specific models. Significant species differences (e.g., human-specific Alu elements and NEAT1_2 lncRNA) limit the utility of commonly used rodent models in simulating RNA editing-related pathologies (Belur et al., 2024). Future research may increasingly rely on iPSC-derived human neuronal models, brain organoids, and humanized mouse models to more accurately simulate pathological processes and evaluate the efficacy of candidate interventions.

Fourth, developing precise delivery and regulatory tools. Technological advances are needed to develop delivery vectors capable of penetrating the blood–brain barrier and achieving cell-type-specific targeting. Simultaneously, priority could be given to developing site-specific editing tools (e.g., ADAR-based guide RNA systems) rather than global regulation of ADAR enzyme activity in order to minimize off-target effects and cell-type-specific functional complexities (Cheng et al., 2025; Li et al., 2025). Approaches such as self-regulating editing complexes (e.g., miRNA-responsive ADAR variants) and optogenetic regulatory systems also warrant further exploration (Li et al., 2025).

Fifth, systematically evaluating the biomarker potential of peripheral blood RNA editing signatures. The potential of peripheral blood RNA editing features as PD biomarkers remains in the early exploratory stage, and their clinical validation requires addressing several fundamental issues. A primary challenge lies in the unclear brain-peripheral association—significant differences exist between the editing profiles of brain tissue and peripheral blood (Song et al., 2023). Directly using peripheral editing features to infer intracerebral pathology may be overly simplistic, necessitating empirical validation through autopsy paired samples or brain-derived exosome technology. Second, multiple confounding factors (age, medications, systemic inflammatory status, changes in blood cell composition) may interfere with editing signals, and their impacts have not been systematically assessed. Additionally, existing studies predominantly employ cross-sectional designs, with insufficient validation of longitudinal stability, diagnostic specificity, and cross-cohort reproducibility (Wu et al., 2023; Li et al., 2024). Finally, the absence of standardized detection and analysis protocols limits comparability across study results. Therefore, the current focus should be placed on systematically addressing these fundamental questions rather than rushing clinical translation. Premature application of immature technologies could undermine long-term prospects in this field due to issues arising from false-positive or false-negative results.

Sixth, extending investigations to other epitranscriptomic mechanisms. RNA editing is merely one component of the epitranscriptomic regulatory network. Future research could integrate RNA editing with other post-transcriptional modifications (such as m6A methylation and non-coding RNA regulation) to more comprehensively understand the complexity of gene expression regulation in PD (Li et al., 2024).

Seventh, explore the potential role of RNA editing in non-motor symptoms (speculative direction). Although there is currently no direct evidence indicating a causal relationship between abnormal RNA editing of 5-HT receptors and non-motor symptoms in PD (such as depression and anxiety), based on the known functions of this pathway in other diseases or models, as well as the high incidence of non-motor symptoms in PD patients, this direction is worthy of direct verification through PD-specific samples (such as different regions of brain tissue, paired behavioral assessments) in future research. Currently, it should be regarded as an exploratory hypothesis rather than an established mechanism.

## 5 Discussion

Collectively, the evidence reviewed here indicates that A-to-I RNA editing is reproducibly dysregulated in PD, but the direction and magnitude of changes are highly context-dependent: brain tissue consistently shows decreased exonic editing and reduced ADAR expression, while peripheral blood shows more variable patterns and patient-derived iPSC dopaminergic neurons show increased editing via ADAR3 downregulation. Mechanistic pathways linking editing to PD pathology vary markedly in evidence strength—strong support exists for SFPQ/NONO dysfunction (with a suggested causal hierarchy from SNCA mutations to nuclear RNA retention) and ADAR1-mediated neuroinflammation (though whether this drives or compensates for pathology remains unresolved), while evidence for mitochondrial and glutamate receptor pathways is moderate, and direct PINK1/Parkin editing remains speculative. A critical conceptual distinction—often blurred in the literature—is that between endogenous editing dysregulation as a potential disease mechanism and engineered editing as an experimental therapeutic tool. Key unresolved uncertainties include causal direction (drivers vs. compensators vs. epiphenomena), cell-type specificity (bulk tissue and purified neurons show opposing signals), brain-periphery correlation (whether blood signatures reflect brain pathology is unknown), and model system generalizability (mice lack human-specific Alu elements). Thus, while RNA editing in PD represents an exciting and mechanistically plausible area of investigation, the field remains largely in the association phase; future priorities should shift from descriptive studies toward causal validation, cell-type-resolved analyses, and rigorous clinical translation efforts.

## 6 Epilogue

RNA editing has opened new horizons in the study of PD. However, transitioning from “correlation” to “causality” and from “lab discoveries” to “clinical translation” will require cautious advancement by the entire research community. Maintaining a clear-eyed assessment of evidence strength, acknowledging the limitations of model systems, and adhering to rigorous causal inference in mechanistic studies—only through these measures can RNA editing research aspire to deliver genuine clinical benefits for PD patients.
